# A Rare Double Heterozygous Mutation in Low-Density Lipoprotein Receptor and Apolipoprotein B-100 Genes in a Severely Affected Familial Hypercholesterolaemia Patient

**DOI:** 10.7759/cureus.12184

**Published:** 2020-12-20

**Authors:** Lilla Juhász, István Balogh, László Madar, Beáta Kovács, Mariann Harangi

**Affiliations:** 1 Division of Metabolism, Department of Internal Medicine, University of Debrecen Faculty of Medicine, Debrecen, HUN; 2 Division of Clinical Genetics, Department of Laboratory Medicine, University of Debrecen Faculty of Medicine, Debrecen, HUN; 3 Department of Internal Medicine, University of Debrecen Faculty of Medicine, Debrecen, HUN

**Keywords:** double heterozygous, autosomal dominant hypercholesterolaemia, familial hypercholesterolaemia, phenotype

## Abstract

Familial hypercholesterolaemia (FH) is characterized by high plasma low-density lipoprotein cholesterol (LDL-C) levels and premature cardiovascular disease risk. Mutations in the genes that encode proteins involved in LDL uptake and catabolism, including LDL-receptor (LDLR) and apolipoprotein-B (APOB), are known to cause FH. We present the case of a severely affected FH proband with two mutations in two different causing genes and characterize her first-degree blood relatives. The proband was a 54-year-old woman with a severe FH phenotype with treated LDL-C of 8.3 mmol/L, total cholesterol (TC) level of 11.6 mmol/L, peripheral artery disease, early myocardial infarction, aortic stenosis, and carotid artery disease. Exons of the LDLR and APOB genes were amplified by polymerase chain reactions (PCR). PCR products were examined by pyrosequencing and proven by bidirectional DNA sequencing. The proband was heterozygous for both the LDLR c.420G>C (p.Glu140Asp) mutation known to be pathogenic and a rare APOB c.10708C>T (p.His3570Tyr) mutation with unproven pathogenicity. Cascade testing has been performed in her 15 first-degree blood relatives. Her daughter carries only the LDLR c.420 G>C mutation with a TC of 8.4 mmol/L. Her two sisters carry only the APOB c.10708C>T with a TC of 5.7 and 6.2 mmol/L. This case provides evidence that the rare APOB c.10708C>T mutation alone is not pathogenic, but has a synergic effect on LDLR mutation. The finding is important for understanding the genotype-phenotype correlation and highlights the need to consider the presence of additional mutations in FH families where relatives have varying phenotypes.

## Introduction

The term familial hypercholesterolaemia (FH) is commonly used to refer to an autosomal dominant gene disorder characterized by increased plasma levels of total cholesterol (TC) and low-density lipoprotein cholesterol (LDL-C), with tendon xanthomas and premature symptoms of coronary heart disease (CHD) [1]. Indeed, the disorder is known to be caused by mutations in at least three different genes [2]. A preferred general term is autosomal dominant hypercholesterolemia (ADH), with FH being reserved for the most common form of the disease due to loss-of-function mutations in the low-density lipoprotein receptor (LDLR) gene, which is responsible for hepatic clearance of low-density lipoprotein (LDL) from the blood circulation, and the disease is also known as ADH-1 [3]. ADH can also be caused by loss-of-function mutations in the apolipoprotein B-100 (APOB) gene, which encodes the ligand for LDLR; in this case, the disease should be referred to as familial defective APOB (FDB) or ADH-2 [4]. Expression of the proprotein convertase subtilisin/kexin type 9 (PCSK9) normally downregulates the LDLR pathway by indirectly causing degradation of LDLR protein. In some of the cases, gain-of-function mutations are present in the PCSK9 gene, which may be referred to as PCSK9-related ADH or ADH-3 [4]. Recently, a novel ADH locus at 4p13 has been identified. STAP1, the encoding signal transducing adaptor family member 1, is the fourth gene associated with ADH [5]. Additionally, the existence of further pathologic mutations in other unknown genes is also probable.

ADH patients are characterized by elevated LDL-C levels, which result in excess deposition of cholesterol in tissues, leading to accelerated atherosclerosis and increased risk for premature CHD [4]. All the monogenic forms of ADH exhibit a gene dosage effect with the severest phenotypes observed in homozygous and compound or double heterozygous carriers, who may, if left untreated, already develop overt CHD in their second decade of life [6,7]. As a result of high cholesterol levels, tendon xanthomas, xanthelasma, and corneal arcus may also occur [8].

It has been shown that LDL-C levels vary greatly among ADH patients [9], which is partly related to the gene, i.e., LDL-C levels in gain-of-function PCSK9 mutation carriers are generally higher compared to loss-of-function LDLR and APOB mutation carriers [10], partly due to the type of gene defect (e.g., deficient versus defective mutations) and other not completely understood phenomena [9]. Moreover, the difference in cardiovascular risk must be largely due to environmental differences as well [11].

Though the clinical consequences of heterozygous and homozygous mutations in one of the ADH-causing genes have been described in great detail, little is known about the phenotypes of ”double-heterozygous carriers”, which are a combination of a mutation in LDLR and APOB or in LDLR and PCSK9 genes [6]. Indeed, besides providing a better understanding of the genotype-phenotype correlation, detection of these variants may have therapeutic consequences as well. Moreover, in some of the cases, the pathogenicity of the identified mutations is still unproven or debated. 

Therefore, our aim was to describe a double heterozygous variant with a known pathogenic mutation in the LDLR gene and with a rare APOB gene mutation with unproven pathogenicity in a severely affected FH patient.

## Case presentation

The sample collection and the study was performed in accordance with the Research Ethics Committees regulations at the University of Debrecen, and all subjects gave informed consent. The characteristics of the studied family are summarized in Table 1.

**Table 1:** Characteristics and lipid profile of the studied family FM: female; M: male; TC: total cholesterol; LDL-C: low-density lipoprotein cholesterol; HDL-C: high-density cholesterol; TG: triglyceride; CHD: coronary heart disease

Variables															
Sex	FM	M	M	FM	FM	M	FM	FM	M	FM	FM	M	FM	M	FM
Age (years)	62	33	38	59	65	41	42	24	22	27	25	23	50	52	70
TC (mmol/L)	12.7	6.2	5.4	6.2	5.7	5.4	8.4	5	4	5.2	5.6	3.3	5	7.2	6.8
LDL-C (mmol/L)	8.3	3.1	3	4.1	3.2	2.6	6.3	2.6	2.1	3.5	3	1.6	3	4.6	3.5
HDL-C (mmol/L)	1.2	1.7	1.9	1.6	1.9	2.3	1.7	1.7	1.1	0.8	1.9	1.2	1.1	0.9	1.3
TG (mmol/L)	1.9	4.8	1.9	1.3	1.2	0.7	0.7	0.9	2.1	2.2	0.8	1	1.9	5.9	5.2
Family history of CHD	+	+	+	+	+	+	+	+	+	+	+	+	+	+	+
Genetic analysis	LDLR + APOB	APOB	APOB	APOB	-	-	LDLR	-	-	-	-	-	-	-	-

Our female proband was referred to the Lipid Outpatient Clinic at the age of 54 because of a TC level of 11.6 and an LDL-C level of 8.6 mmol/L despite receiving the maximum dose of combined lipid-lowering therapy (rosuvastatin 40 mg + ezetimibe 10 mg/day). In her medical history, premature CHD, carotid artery disease, aortic stenosis, and peripheral artery disease (PAD) were found. At the age of 52, she had undergone a successful percutaneous coronary intervention due to acute myocardial infarction. At the age of 53, a successful iliofemoral bypass surgery had been done due to critical ischemia of the left lower extremity. During the physical examination, xanthelasma was found as an external manifestation of FH. The Dutch Lipid Clinic Network criteria are one of the most widely used scoring systems for the diagnosis of FH [1]. Our patient’s score was 21; therefore, her FH diagnosis was based on the anamnestic, clinical, and laboratory data. However, the genetic examination was still deemed reasonable because of the extremely high lipid parameters despite the combined lipid-lowering therapy and the severe premature cardiovascular manifestations to exclude the homozygosity and compound or double heterozygosity, which may alter the therapeutic strategy. Based on their higher prevalence, LDLR and APOB genes were examined first. Furthermore, cascade testing had been performed in her 15 first-degree blood relatives. In all studied cases, secondary causes of hypercholesterolaemia were excluded based on their anamnestic data, physical examination, and routine laboratory test.

Venous blood samples were taken after an overnight fast and sera were prepared immediately. Routine lipid analyses [TC, LDL-C, triglyceride, and high-density lipoprotein cholesterol (HDL-C)] were performed from fresh sera with Cobas c501 autoanalyzer (Roche Ltd., Mannheim, Germany) in the Department of Laboratory Medicine of the University of Debrecen. Reagents were purchased from the same vendor and the tests were performed according to the recommendation of the manufacturer.

Genomic DNA was isolated from ethylenediaminetetraacetic acid (EDTA) or citrate-anticoagulated blood using QIAamp Blood Mini Kit (Qiagen GmbH, Hilden, Germany). Part of exon 26 of the APOB gene where the known mutational hotspot is located was amplified by polymerase chain reaction (PCR) process and sequencing by bidirectional Sanger sequencing method. The coding region of the LDLR gene was amplified by separate PCR processes and the products were examined by pyrosequencing using GS Junior Titanium Sequencing Kit and GS Junior DNA sequencer (Roche 454 Life Sciences, Branford, CT). Data were analyzed using the GS Amplicon Variant Analyzer 2.9 software. The detected variant was confirmed by Sanger sequencing. In the case of APOB, the current nomenclature was followed.

The LDLR gene analysis proved that our proband is heterozygous for the LDLR c.420 G>C (p.Glu140Asp), which is a known pathogenic mutation [12]. The APOB gene analysis showed that the patient is also heterozygous for a rare APOB c.10708C>T (p.His3570Tyr) mutation with unproven pathogenicity. The first description of this mutation assumed its pathogenicity [13]. However, a later publication mentioned it as a harmless polymorphism [14].

**Figure 1:**
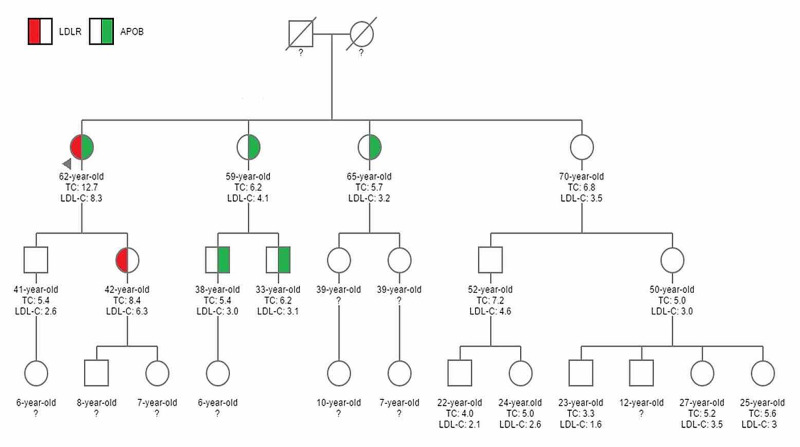
Pedigree of the studied family LDLR: low-density lipoprotein receptor; APOB: apolipoprotein B; TC: total cholesterol; LDL-C: low-density lipoprotein cholesterol

In order to prove or to exclude the pathogenicity of the rare APOB mutation, cascade testing has been performed in 15 first-degree blood relatives of the proband (Figure 1).

The daughter of the proband carries only the LDLR c.420 G>C mutation with a TC of 8.4 mmol/L. The son of the proband is wild-type for both mutations with a TC of 5.4 mmol/L. Two sisters of the proband carry only the APOB c.10708C>T mutation with a TC of 5.7 and 6.2 mmol/L.

## Discussion

Structural abnormalities caused by mutations in the receptor-binding domain of the APOB gene may lead to defective binding of LDL particles to LDL receptors. Soufi et al. screened the APOB gene segment of codons 3448-3561 (3475-3588 using the current nomenclature) in 853 German patients who underwent diagnostic coronary angiography for suspected CHD. By this, a novel single base mutation, p.His3570Tyr mutation, was detected. The prevalence of heterozygotes for p.His3570Tyr in the study population was 0.47% compared to 0.12% for the known pathogenic mutation p.Arg3500Gln (p.Arg3527Gln using the current nomenclature). In short, the new mutation was four times more frequent than “classical” FDB and thus appeared to be the most common APOB mutation in Germany [13].

In a later study, Basistová et al. investigated the frequency of the p.His3570Tyr mutation in 362 probands who fulfilled the clinical criteria for the diagnosis of FH. The prevalence of the p.His3570Tyr mutation in their group of unrelated subjects with FH phenotype was 0.276%, which was 48.5 times less frequent than the prevalence of the p.Arg3500Gln mutation. The low frequency of this mutation in probands with FH phenotype, as well as its distribution in the families of affected probands, indicates that this mutation has a minor effect on LDL-C levels. Therefore, they supposed that the p.His3570Tyr is not a pathogenic mutation, but a polymorphism in the APOB gene [14]. This controversy still exists. The Human Gene Mutation Database (HGMD), version 2017.4 describes it as a disease-causing mutation, while the prediction tools Mutation Taster and Polyphen categorize it as "polymorphism" and "benign", respectively. In the Exome Aggregation Consortium (ExAC) database, the mutation is present with very low, 0.0001485 allele frequency. According to the ClinVar, its clinical significance is "Conflicting interpretations of pathogenicity" (evaluated on Jun 16, 2017).

Our results provide evidence that the rare APOB c.10708C>T mutation alone is not pathogenic. However, the combined effect of an LDLR and an APOB mutation leads to a phenotype more severe in terms of atherosclerotic vascular diseases than either mutation alone. The limitation of this study should also be mentioned. Unfortunately, we could not measure LDL receptor function directly because of technical difficulties and costs.

More than 1,700 different variants of the LDLR gene were found to cause FH, making genetic screening very laborious [15]. In contrast, only a few pathogenic mutations of APOB and PCSK9 were found and their causative role is often unproven [16,17].

Clinical criteria most commonly used for diagnosis of FH include the Dutch Lipid Clinic Network criteria [1]. Major items for these clinical diagnostic criteria are severe hypercholesterolaemia, physical findings such as xanthoma in the index case, and a family history of premature CHD. For a cost-effective and efficient diagnosis, many guidelines and expert groups recommend cascade genetic screening for family members. However, in everyday practice, diagnosis using only clinical findings from history taking, physical examination, and lipid profile is very common, especially when genetic testing is not available [17].

To date, there is no cure for FH. The primary goal of clinical management in severely affected patients is to control hypercholesterolaemia in order to decrease the risk of atherosclerosis and to prevent or delay cardiovascular manifestations [16]. The most commonly used lipid-lowering drugs are statins, ezetimibe, bile acid resin, and fibrates [17]. However, even after treatment with combination therapy, the majority of severely affected FH patients may still have elevated LDL-C levels [18], and their risk of CHD remains unacceptably high [15]. There are some novel therapeutic options including microsomal triglyceride transfer protein inhibition, an antisense oligonucleotide against APOB RNA (approved only for homozygous patients), and monoclonal antibodies against PCSK9 (approved for both heterozygous and homozygous patients). Several further therapeutic approaches are under development, such as squalene synthase inhibitors, siRNA for PCSK9 or for APOB silencing, and antisense PCSK9. Moreover, selective LDL apheresis is available for severe heterozygous and homozygous patients [19]. Therefore, in selected cases, genetic analysis is essential to administrate the most effective therapeutic options mentioned above.

## Conclusions

Our proband with a double heterozygous mutation had higher LDL-C levels and severe premature CHD. This case provides evidence that the rare APOB c.10708C>T mutation alone is not pathogenic. However, the combined effect of an LDLR and an APOB mutation leads to a phenotype more severe in terms of atherosclerotic vascular diseases than either mutation alone, indicating that the sum of the genetic burden needs to be considered. It also highlights the need to consider the presence of additional mutations (APOB, PCSK9, STAP1) in families with FH where relatives have varying phenotypes to find the most appropriate therapeutic strategy for our patients.
